# Integrating health humanities in medical education: a narrative scoping review of Mazandaran University of Medical Sciences and global perspectives

**DOI:** 10.1186/s13010-026-00216-3

**Published:** 2026-05-01

**Authors:** SeyedAmir Mohseni, Siavash Moradi, Fattane Amuei

**Affiliations:** Mazandaran University of Medical Sciences, Sari, Islamic Republic of Iran

## Abstract

The medical humanities are an interdisciplinary field that combines the humanities, social sciences, and arts with medical education and practice. It fosters a multidisciplinary understanding of the human condition, patient suffering, the roles of healthcare professionals, and the community surrounding them. Familiarity with the humanities enhances physicians’ perspectives, communication skills, understanding of patients, and overall quality of healthcare. This article reviews the general and health humanities courses offered at the Faculty of Medicine at Mazandaran University of Medical Sciences (MAZUMS) in Iran, and those available worldwide. New incremental courses have been introduced at MAZUMS, along with early-stage integrated approaches. Our study of universities worldwide examined medical schools across the US, Canada, Europe, the UK, China, Taiwan, India, Nepal, Sri Lanka, Africa, and Australia. It aimed to understand how health humanities are organized, their importance, and their benefits. We found that while Mazandaran University of Medical Sciences recently added health humanities courses, integration remains limited compared to leading institutions. The study demonstrates that incorporating health humanities enhances empathy, reflective skills, and patient care. These findings suggest that a structured approach can fill gaps, improve medical education in Iran as well as many countries, and align it with global standards.

## Introduction

The medical humanities are an interdisciplinary field that combines the humanities, social sciences, and arts, along with their applications in medical education and practice [1]. It offers an approach that fosters a deeper understanding of the human condition, suffering, patients, health professionals, and their communities [2]. Familiarity with the humanities in medical education enables physicians to approach their work with a broader perspective, connect more effectively with patients, understand their perspectives, and deliver higher-quality healthcare [1].

Topics covered in medical/health humanities include understanding disease from the patient’s perspective, empathy, and establishing a strong doctor-patient relationship (rapport), understanding the patient’s narrative of illness, making medical practice more humane, increasing resilience in physicians, enhancing creativity and emotional understanding, and addressing medical ethics issues such as euthanasia, technology-driven medicine, and futures studies [3].

In Iran, interdisciplinary topics are still emerging, with an increasing focus in scholarly works over the past decade. The medical humanities, as an interdisciplinary field, faces gaps between the humanities and the medical sciences [4]. Few studies have explored the role of the humanities in medicine; several studies suggest that it enhances physicians’ empathy, reflection, recovery, health, and resilience [5]. Scholars, including Ledger and Joynes [6], Wu and Chen [7], and Kalateh Sadati and Bagheri Lankarani [8], investigate this interaction, suggesting that engagement facilitates dialogue and knowledge exchange, thereby shaping and expanding the field of medical humanities to become a science. A study at Shahid Sadoughi University of Medical Sciences in Yazd emphasizes the development of these fields through institutional, cultural, and policy efforts [9].

The results of a study at the Berlin Medical School highlighted the social aspects of the patient-physician relationship and the medical profession. This study demonstrated that integrating the humanities into medical education enables students to enhance their understanding of the strengths and limitations of modern medicine, cultivate a sense of responsibility, and broaden their perspective and worldview [10].

Despite the potential benefits of the humanities in medical education for individual doctors and the medical community as a whole, there are fundamental gaps between the humanities and the medical sciences. In recent decades, many efforts have been made to include the humanities in medical school curricula, and this trend has gained momentum in recent years. Various methods, including classroom lectures, observation of empathetic behavior, role-playing, studying patient narratives of illness, reading excerpts from classic medical texts, and analyzing audio or video recordings of patients’ encounters with healthcare professionals, have been proposed for teaching humanities in medicine. Still, evidence suggests that there are significant barriers to maintaining these methods in the future [8].

The most effective approach is to incorporate and integrate the humanities into the general medical curriculum; as a result, many of the world’s leading universities now include medical humanities in their curricula. A review of international and limited domestic studies shows most focus on the humanities’ role in medical education or adding related courses. None address organizing, integrating, and structuring medical humanities within curricula to reflect its interdisciplinary nature. Globally, numerous reviews and studies have examined approaches to this integration, primarily providing broad frameworks that lack a focus on specific countries or institutional contexts. No comprehensive national study, especially at Mazandaran University of Medical Sciences, has been done. This study aims to fill that gap, describing MAZUMS’ situation and comparing it with global efforts to inform future policies.

### Origins of medical humanities

The concept of medical humanities in the United States emerged in the twentieth century as a response to a fundamental philosophical question: What is medicine or the practice of medicine? During this period, medicine was closely linked with science. Scientific or Western medicine became widely accepted, and its strong influence often leads to viewing medical curricula as science-based disciplines [11]. However, the need to see medicine as more than just science—something more complex—has been recognized for a long time. The respected US physician Francis W. Peabody believed that practicing medicine should involve a comprehensive and holistic relationship between doctors and patients, as medicine is an art that is increasingly grounded in science but also includes aspects beyond scientific explanation [12].

The rapid growth of knowledge and technology has expanded the scope and effectiveness of medicine, but it has also created new challenges, such as the overmedicalization of human conditions [13]. As technology continues to develop, promoting the humanities becomes essential because there is concern in both medical education and practice that technology might overshadow traditional human values by focusing too much on scientific aspects. Therefore, medical practice and clinical medicine need to consider questions of values, including ethical considerations. Pellegrino clearly sees this importance and warns that the gap between technology and human values poses the greatest danger in medicine. When the focus is solely on humans and the human condition, while science is emphasized at the expense of human values and dimensions, it can lead to dehumanizing patient care [14].

In his book “Humanism and the Physician” [14], Pellegrino examines the idea of medical humanism, emphasizing that medicine involves more than just science and technique — it also includes a human, ethical, and existential relationship between the doctor and the patient. He argues that healthcare providers should focus on the patient’s humanity, dignity, and values in clinical practice, rather than only addressing the physical body or illness.

His book covers key topics such as the physician’s moral responsibility, mutual trust, the meaning of suffering, and the role of human values in clinical decision-making. Pellegrino highlights that medicine loses its meaning without a human and ethical dimension, reducing it to mere technology. He advocates for incorporating the humanities (philosophy, ethics, history, literature) into medical education and practice, asserting that ignoring these aspects turns medicine into just “technique”. He believes that understanding concepts like suffering, trust, the doctor-patient relationship, and moral responsibility requires a humanistic and interdisciplinary approach, which is now called the medical humanities. Ultimately, he connects medicine as a biological science with it as a human art—a perspective that later influenced the development of the field of medical humanities.

### Global relevance and contribution of this review

Although this review focuses on the integration of health humanities at Mazandaran University of Medical Sciences, its relevance extends beyond a single institutional context. Many medical schools around the world, particularly in low- and middle-income countries, are currently exploring how to integrate the humanities into predominantly biomedical curricula while addressing cultural, educational, and institutional limits. The review of the MAZUMS’ experience provides a valuable case study that illustrates both the opportunities and challenges of implementing health humanities in a developing educational context. It should also be noted that, based on the evidence available as of March 2026, the approved general medical curriculum courses are presented in many Iranian medical schools in much the same way.

For international readers, this review provides comparative insights into curriculum design, cultural adaptation of medical humanities education, and strategies for integrating humanistic perspectives into clinical education. It also highlights transferable lessons about faculty development, curriculum structure, and the potential educational impact of humanities-based approaches. These insights may inform policymakers, educators, and curriculum planners seeking to strengthen the humanistic dimensions of medical education in diverse global settings.

Examining the development of medical humanities in a specific institutional context, such as Mazandaran University of Medical Sciences, provides insight into how humanities-based education is adapted to different socio-cultural and educational environments. Such case analyses contribute to the global discourse on the localization and contextualization of medical humanities curricula.

### Objectives of the review

The main objective of this narrative scoping review was to examine the current status, structure, and integration of health humanities in the undergraduate medical curriculum of Mazandaran University of Medical Sciences. The review also aimed to compare these developments with international experiences in medical humanities education to identify similarities, differences, and existing gaps. Another objective was to examine the reported educational impacts of health humanities on medical students. Finally, the review seeks to provide evidence-based insights that can guide curriculum development, policy planning, and the future integration of medical/health humanities in medical education.

## Methodology

This study conducted a narrative scoping review to examine the structure, integration, and delivery of medical humanities in undergraduate curricula. The choice of a narrative scoping review was made because of the broad and multifaceted nature of the literature. Unlike systematic reviews, which are suited for narrow, homogeneous data, health humanities are diverse, with variations in methodologies, settings, and focus areas. A scoping review helped compile, map evidence, identify themes, and find gaps without excluding sources based on methods. The narrative approach provided context for results at both national and global levels, allowing for comparison and reflection.

A thorough review was carried out using international databases, including PubMed, Scopus, and Google Scholar, as well as national Persian literature repositories. Keywords such as Medical Humanities, Health Humanities, Medical Education, and Humanities in the Medical Curriculum were used during the search. Articles were selected based on their relevance to humanities instruction in medicine, excluding those that focus solely on non-medical careers. An effort was made to include research from various countries and cultures to enable comparative insights. Following data collection and analysis, the findings were organized qualitatively and thematically to compare with universities in other countries.

### Search strategy

The literature search was conducted using several international databases, including PubMed, Scopus, and Google Scholar. As an example, the following search strategy was used in Google Scholar:

(“medical humanities” OR “health humanities”)

AND (“medical education” OR “medical curriculum” OR “medical students”).

AND (integration OR teaching OR education).

The search was limited to articles published in English or Persian between 2000 and 2025. Reference lists of relevant studies were also screened to identify additional publications.

### Inclusion/exclusion criteria

Inclusion criteria included: (1) articles about medical or health humanities in undergraduate medical studies; (2) articles written in Persian or English; and (3) articles published between 2000 and 2025.

Reasons for exclusion included: (1) articles related to broad humanities that cannot be connected to medical teaching; (2) manuscripts with insufficient methodological or curricular information; and (3) opinion articles or commentaries lacking an empirical basis or a formal review framework.

The selection process involved three stages: (1) initial identification of records through keyword searches; (2) screening of titles and abstracts to exclude irrelevant studies; and (3) full-text review for final eligibility. After applying the inclusion and exclusion criteria, studies were included in the final analysis.

Data from the studies were narratively summarized based on curriculum design, integration, course content, student outcomes, and implementation issues. Findings were thematically categorized to gain insights and compare MAZUMS’ curriculum.

### Review process and author roles

This review was conducted as part of an MD thesis project. The primary author performed the literature search, screening of studies, data extraction, and initial drafting of the manuscript. Two academic supervisors were involved in the process: one supervisor actively contributed to the development of the review, including methodological guidance, interpretation of findings, and manuscript preparation, while the second supervisor provided overall supervision, critical feedback, and assistance in resolving methodological or conceptual issues throughout the study. Regular consultations ensured consistency and scientific rigor during the review process.

## Results

### Study selection process

A comprehensive literature search was conducted across several international databases, including PubMed, Scopus, and Google Scholar, to identify studies relevant to the integration of medical humanities in medical education. The search yielded a large number of potentially relevant records. After the removal of duplicate entries, titles and abstracts were screened to assess their relevance to the objectives of the review.

Articles that appeared to meet the inclusion criteria were subsequently retrieved for full-text assessment (*N* = 78). During this stage, studies were evaluated based on predefined inclusion and exclusion criteria related to topic relevance, educational context, and publication language.

Following the eligibility assessment, a total of 34 studies were considered suitable and were included in the final qualitative synthesis.

The selection process followed a structured and iterative approach consistent with established review methodologies. Any uncertainties regarding study eligibility were resolved through discussion among the research team to ensure methodological rigor and consistency in study selection.

### Characteristics of included studies

The included literature demonstrated substantial methodological diversity. Conceptual and theoretical papers, including classical essays, constituted the largest proportion of the sources (*N* = 11, 32%), reflecting the normative and philosophical foundations of medical humanities scholarship. Among empirical studies (*N* = 23, 68%), qualitative approaches were most common (*N* = 7, 21%), followed by review studies (*N* = 6, 18%), descriptive reports of curriculum development (*N* = 5, 15%), and educational intervention studies evaluating the impact of humanities-based teaching strategies (*N* = 4, 12%). A smaller number of studies employed cross-sectional survey designs.

The included studies were mostly published between 2000 and 2025 and represented diverse geographical regions, including America, Asia, Africa, Europe, and Australia.

Most studies employed qualitative, descriptive, review, interventional, and mixed-methods designs and primarily focused on undergraduate medical students. The main thematic areas identified across studies included:

- Curriculum design and integration models.
- Educational outcomes and student experiences.
- Assessment of empathy and professionalism.
- Reflective practice and narrative medicine.
- Arts-based and experiential learning approaches.

The detailed characteristics of the included studies are summarized in Table 1.

**Table 1:** Characteristics of the included studies

Author	Year	Country/Region	Main Focus	Key Findings
Blease	2016	UK	Philosophical discussion	Defended educational value of humanities for medical students
Bekele et al.	2024	Africa	Integration of liberal arts in medical curriculum	Emphasized foundations in social medicine, critical thinking, and community-based education; improved health equity awareness.
Kosik et al.	2018	China	Role of medical humanities in Chinese medical schools	Identified 8 types of courses (e.g., medical psychology, ethics); humanities comprise 3–10% of curriculum; mandatory ideological courses add 6–8%.
Tsai	2008	Taiwan	Community-oriented curriculum design	Improved communication skills, confidence, and social trust through community service; fostered self-learning and empathy.
Shankar	2016	India	Institution-driven MH initiatives	Used small-group activities (narrative writing, role-plays); balanced standardization with cultural adaptation; enhanced empathy and reflection.
Joshi et al.	2018	India	Structured integration with assessment	Incorporated narrative medicine, arts; used Jefferson Scale of Empathy; improved empathy, ethics, and professionalism.
Shankar et al.	2011	Nepal	Use of paintings and reflective exercises in MH modules	Addressed empathy, doctor-patient relationships; feasible in South Asian contexts despite faculty and time constraints.
Jayasinghe & Fernando	2023	Sri Lanka	Longitudinal integration with arts and narratives	Incorporated local cultural traditions; fostered empathy and professionalism; established Department of Medical Humanities.
Jones & Verghese	2003	USA	Longitudinal humanities curriculum development	Focused on ethics, reflection, and arts; improved critical thinking, cultural sensitivity, and communication skills.
Kiessling et al.	2003	Germany	Special study modules on medical theory	Enhanced awareness of medicine’s strengths/weaknesses; broadened worldview and sense of responsibility.
Ledger & Joynes	2018	UK	Music engagement	Supported identity formation and emotional development.
Coronado-Vázquez et al.	2023	Europe	Evaluation of humanities curricula outcomes	Improved empathy, ethical reasoning, and reflective capacity; emphasized epistemological justification.
Dhara & Fraser	2024	Canada	Health humanities for advocacy skills	Used narratives and arts; improved communication, self-reflection, and patient-centered advocacy.
Chiavaroli & Ellwood	2012	Australia	Critical discussion of integration	Warned against superficial integration
Melhem et al.	2025	Australia	Professional practice program with reflection	Enhanced communication and leadership; challenges in student perceptions of humanities vs. biomedical sciences.
Wald et al.	2019	Global	Integration of arts and humanities in education	Fostered empathy, resilience, professional identity and well-being; complemented clinical training.
Graham et al.	2016	USA	Impact on empathy using Jefferson Scale	Elective courses associated with improved empathy scores; mitigated empathy decline.
Jones et al.	2017	USA	Creative arts-based learning	Enhanced reflection, empathy, and identity development.
Salajegheh	2022	Iran	Conceptual discussion of humanities education	Highlighted benefits for ethics, empathy, and professionalism.
Mohseni et al.	2024	Iran	Integration of Health humanities in MD curriculum	Supported by the faculties. Challenges and approaches were identified.

### Global models of health humanities integration

Analysis of the literature revealed several models for integrating health humanities into medical education:

1. **Stand-alone compulsory courses**: offered during pre-clinical training.
2. **Elective humanities modules**: allowing students’ choice.
3. **Longitudinal integration models**: embedding humanities themes across multiple years.
4. **Experiential and arts-based approaches**: including literature, visual arts, narrative writing, and theatre as a pedagogical approach.

While high-income countries often implemented longitudinal and structured integration models, several low- and middle-income settings adopted more limited or elective-based approaches.

A comparative overview of these integration models is presented in Table 2.

**Table 2:** A comparative overview of integration models and teaching approaches

Integration Model	Example Countries/Regions	Key Features	Advantages	Challenges
Stand-alone Compulsory Courses	China, India, Iran, USA	Dedicated courses in the pre-clinical phase such as medical ethics, medical sociology, psychology, or history of medicine; usually assigned fixed curricular credits.	Ensures all students receive foundational humanities exposure; easy to implement and assess.	Limited integration with clinical training; may feel disconnected from practical medicine; risk of being seen as peripheral or “add-on” rather than core components.
Elective Humanities Modules	Nepal, Sri Lanka, Australia, Canada	Optional modules including narrative medicine, literature, visual arts, reflective writing, or philosophy of medicine; typically offered as electives or extracurricular activities.	Encourages student motivation and self-directed engagement; allows exploration of diverse interests and creative learning approaches.	Participation may be limited to interested students only; unequal exposure among students; sustainability may depend on faculty interest and institutional resources.
Longitudinal Integration Models	Germany, UK, USA, Taiwan	Humanities themes integrated across multiple phases of medical education (pre-clinical and clinical years); includes continuous reflective practice, ethics discussions, and patient-centered narratives.	Promotes progressive development of empathy, ethical reasoning, and professional identity; aligns humanities education with clinical learning experiences.	Requires extensive curriculum coordination and institutional commitment; evaluation of long-term outcomes may be methodologically complex.
Experiential and Arts-based Approaches	India, Nepal, Sri Lanka, Africa	Experiential learning activities such as role-plays, theatre, storytelling, reflective writing, visual arts, and community engagement projects.	Enhances communication skills, cultural competence, and emotional awareness; adaptable to diverse cultural contexts and learning environments.	Resource-dependent; learning outcomes may be difficult to standardize and objectively measure; requires faculty training in interdisciplinary teaching methods.

### Reported educational impacts of health humanities

Across the included studies, health humanities education was associated with several reported outcomes among medical students:

- Increased empathy and patient-centered attitudes.
- Improved communication skills.
- Enhanced reflective capacity.
- Greater awareness of ethical and professional issues.
- Improved tolerance of ambiguity, emotional resilience, and professional satisfaction.

However, variations in study design, assessment tools, and follow-up duration limited direct comparison of outcomes across studies.

### Educational context and curriculum structure at MAZUMS

Mazandaran University of Medical Sciences (MAZUMS), located in Sari, follows the national MD curriculum approved by the Ministry of Health and Medical Education. The program spans approximately 6–7 years and consists of four major phases: Basic Sciences, Physiopathology, Clinical Clerkship, and Internship.

The entire curriculum consists of approximately 293 units of study, which vary slightly across universities and have undergone changes over the years. Within this framework, humanities-related courses account for approximately 30 credits, primarily delivered during the pre-clinical phase. These courses include different subjects, the most important of which are Bioethics, Medical Etiquette, and Health Psychology.

The structure and positioning of humanities within the curriculum reflect both national educational policies and institutional adaptation at MAZUMS. A summary of the humanities-related components at MAZUMS is presented in Table 3.

**Table 3:** A summary of the humanities-related components at MAZUMS

Course	Credits	Hours	Type	Phase	Humanities Focus
Health Psychology	2	34 Theoretical	Mandatory	Basic sciences phase	Psychological understanding of health
Medical Ethics	2	34 Theoretical	Mandatory	Clinical phase	Professional ethics
Medical Etiquette 1–4	2	68 Practical	Mandatory	Basic sciences phase	Communication skills and professionalism
General Humanities Courses	24 total credits	Variable	Mixed, including mandatory and elective courses	Basic sciences phase	General Education, English and Persian Literature, history, culture, theology

## Discussion

### Humanities in MAZUMS curricula

Mazandaran University of Medical Sciences (MAZUMS), located in Sari, follows the national undergraduate medical curriculum framework approved by the Ministry of Health and Medical Education in 2017. The Doctor of Medicine (MD) program in Iran is a continuous six- to seven-year program entered directly after high school through a national entrance examination (Konkour). Admission to the MD program is highly competitive.

The medical curriculum is structured into four main phases: basic sciences (approximately 2 years), physiopathology (1 year), clinical clerkship (2 years), and internship (1.5 years). Each phase includes a defined number of curricular credits determined at the national level.

The Iranian higher education credit system defines one theoretical credit as equivalent to 17 h of instruction per semester, while practical and clinical credits correspond to a higher number of supervised training hours. Humanities-related courses are typically delivered during the pre-clinical years, although some components may be integrated longitudinally throughout the clinical phases. The MD curriculum consists of approximately 293 credits and is structured into four main phases:

1. **Basic Sciences Phase** (approximately 2 years; 93 credits).
2. **Physiopathology Phase** (approximately 1 year; 36 credits).
3. **Clinical Clerkship Phase** (approximately 2 years; 92 credits).
4. **Internship Phase** (approximately 1.5 years; 72 credits).

The total units in the medical program are 293, including 24 units (about 8%) for general-humanities courses. It also includes 69.5 required introductory courses, 177.5 specialized courses, 16 electives, and 6 thesis credits. The general humanities courses conclude with courses in Persian and English Literature, Family and Population Knowledge, Iranian History, the Culture and Civilization of Islam and Iran, as well as courses on Islamic Thought, Theology, and the Islamic Revolution. In addition to these courses, there are also specialized humanities-based courses. These courses include the following:

**Health Psychology** (34 h, 2 credits): A humanities course in basic sciences, aiming to familiarize students with the psychology fields and human psychological traits. It enables understanding of psychological factors in health promotion, quality of life, and disorder prevention.

**Medical Etiquette** Courses 1 to 4 (68 practical hours, 2 credits): Medical Etiquette includes courses designed to empower medical graduates in various areas, including professional behavior in medicine, individual and interpersonal skills, basic principles of learning, personal development, scientific thinking skills, and more. During Medical Etiquette Courses 1 to 4, topics such as learning principles and critical thinking skills, understanding and managing cognitive errors and reasoning, interpersonal communication—including empathy, active listening, and practical expression—patient communication, interprofessional collaboration, and conflict management and problem-solving techniques are taught.

**Medical Ethics** (34 theoretical hours, 2 credits): The medical ethics course, taken early in the internship, covers both human and professional ethics. It aims to help students understand the medical expectations of a competent doctor, recognize ethical issues, and acknowledge their responsibilities. Emphasized attitudes include respect for patient dignity, responsibility, justice, fairness, client rights, and cultural sensitivity.

It is also notable that the course “Literature and Medicine” has been introduced at Tehran University of Medical Sciences. In recent years, MAZUMS has also sought to review its Persian Literature course and make it more compatible with medical students, and to make literature more relevant to medical practice. For example, to add poems and stories related to medicine. However, all these efforts are still at an early stage of integrating Medical and health humanities into the curriculum.

### Regional studies in medical humanities

In 2019, Kalateh Sadati and colleagues conducted a study titled “Analysis of Strategies for Integrating Humanities with Medical Sciences in Iran from the Perspective of Humanities Experts in the Country with the Approach of Convergent Sciences.” The study used a national, qualitative approach carried out in 2019. Considering the saturation criterion, data were collected through 22 semi-structured interviews. The study included professors from several universities and institutes across Iran. Due to limited access to three professors, questions were sent via email as an open questionnaire, and analysis was performed using thematic analysis.

The results showed that participants were critical of the current state of medical humanities in the country, citing a lack of practical or meaningful connection between the humanities and medical sciences. To address this, four themes were identified: institutional development of knowledge, non-institutional development, laws and policies, and culture building. In conclusion, the study suggests that developing medical humanities requires significant national policies, which should be supported by institutions and organizations. These policies should promote closer links between the two fields and emphasize cultural factors. The study also recommends adopting a native approach that revisits the traditions and roots of the humanities, especially the history of literature, in medical education [9].

### Global perspectives on medical humanities

#### Africa

A 2023 article by Abebe Bekele and colleagues titled “Advancing Global Health Equity: The Role of the Liberal Arts in Health Professional Education” states that many initiatives are underway in medical schools across Africa. Although more than half of medical schools in Canada, the United States, and the United Kingdom offer at least one course in medical humanities, this is less common in Africa. A recurring theme was the integration of liberal arts into the medical curriculum, as outlined below.

The first six-month program, called Foundations in Social Medicine, includes courses in critical thinking and communication, African history and global political economy, medical anthropology and social medicine, psychology and health, gender and social justice, information technology and health, and community-based education. Additionally, a research-focused education, characterized by relatively small classes, is demonstrated within an overall institutional culture that emphasizes health equity [15].

#### Asia

In 2018, Kosik et al. conducted a study titled “Medical Humanities Education in China: An Exploratory Cross-Sectional Study.” This study aimed to understand the current role of medical humanities in Chinese medical schools. A cross-sectional, exploratory approach was employed to examine medical humanities education in China. A comprehensive web-based search of records and curricula was conducted to classify all medical humanities courses across Chinese medical schools. In China, only level-one colleges are authorized to provide clinical medical education. All compulsory general curricula of medical schools and their medical humanities programs were included in the analysis. The primary goal was to classify the role of the humanities in Chinese medical schools in terms of both quantity and quality.

All course data were categorized and analyzed using SPSS version 20. The results showed that between July 1, 2017, and April 30, 2018, 138 top-ranked Chinese medical schools with mandatory general curricula were identified, of which 67% offered a medical humanities curriculum. Eight types of medical humanities courses were identified. On average, each school offered 3.84 different types of these courses. The most common were medical psychology or clinical psychology (77%), medical ethics (72%), health or medical jurisprudence (60%), and patient communication (49%). Medical humanities courses comprised 3–10% of the total credits required for medical students to graduate in China. The mandatory ideological and political theory curriculum accounted for 6–8% of graduation credits. This curriculum includes Mao Zedong Thought, modern Chinese history, Marxism, socialism with Chinese characteristics, and moral and legal education, and is required for all students, not just medical students [16].

A study titled Designing a Community-Oriented Curriculum for Medical Humanities was conducted by Tsai (2008) in Taiwan. Recent surveys in Taiwan have shown declining physician satisfaction and increasing frustration with their work environment. Main complaints include stress, long hours, low salaries, disrespect from management, and lack of trust from patients and society. To restore social trust, the study suggests integrating the idea of “physician as an agent in changing the relationship with patients” into the medical curriculum.

The paper argues that structured community service for medical students promotes self-learning and the development of strong clinical and communication skills, while helping students understand that social trust is essential to the practice of medicine. These effects were observed after several years of an experimental curriculum involving over 800 students. A program utilizing community empowerment was implemented in a two-stage curriculum design. Students’ self-assessments showed improvements in communication skills, confidence in expressing their views, effective planning, human resource management, handling limited space and time, and experiencing a deep, dynamic learning process. Therefore, community-based curriculum designs that foster self-learning should be a core part of reforming humanities education in Taiwanese medical schools. Additionally, the medical humanities continue to play a vital role in Taiwan’s ongoing efforts to build a civil society and embed democracy into society [17].

In India, the integration of medical humanities (MH) into undergraduate medical education has developed gradually and remains largely institution-driven rather than centrally mandated. Shankar (2016) notes that MH initiatives in Indian medical schools often rely on small-group, activity-based learning strategies such as narrative and reflective writing, role-plays, art appreciation, debates, and discussions. Rather than imposing a rigid national curriculum, the author suggests that universities provide broad competency guidelines while allowing institutions flexibility to adapt MH modules to local linguistic and cultural contexts. This approach reflects an effort to balance standardization with cultural relevance, while fostering empathy, critical reflection, and structural awareness among medical students [18].

The Indian experience highlights a structured and practice-oriented attempt to integrate medical humanities into undergraduate education. As described by Joshi et al. (2018), humanities are incorporated through diverse modalities, including narrative medicine, poetry, role-playing, cine-education, visual arts, and reflective writing, to cultivate empathy, communication skills, ethical sensitivity, and professional attitudes. Notably, the inclusion of structured assessment methods—such as reflective writing, OSCE-based communication evaluation, and the use of the Jefferson Scale of Empathy—demonstrates an effort to move medical humanities from a hidden curriculum toward a formally assessed and competency-based component of medical training [19].

In Nepal, MH education has largely developed through institution-led initiatives rather than as a nationally mandated curricular component. At KIST Medical College, structured MH modules were conducted for both faculty members and undergraduate medical students, employing interactive and student-centered methods such as small-group discussions, reflective exercises, role-plays, and the use of paintings to stimulate emotional and ethical reflection [20, 21]. The themes addressed included empathy, the doctor–patient relationship, breaking bad news, family roles, and the sociocultural meaning of illness in the Nepalese context. Despite challenges such as limited faculty involvement, time constraints, and the non-compulsory nature of the modules, these initiatives demonstrated that context-sensitive and participatory humanities education is feasible within South Asian medical schools and may positively contribute to professional identity formation and communication skills development [21].

In Sri Lanka, the integration of medical humanities into undergraduate medical education has progressed in a structured and context-sensitive manner. The Faculty of Medicine, University of Colombo—one of the oldest medical schools in Asia—introduced an integrated curriculum in 1995 with longitudinal inputs from Behavioral Sciences, and subsequently established the first Department of Medical Humanities in the country in 2016.

This development marked a shift from a predominantly discipline-based, Flexnerian model toward a more holistic and humanistic framework. The curriculum evolved to incorporate visual and performing arts, narrative-based learning, reflective assignments, and seminars aimed at fostering empathy, compassion, professionalism, and patient-centered care. Initiatives such as the “Humanitas” interdisciplinary webinars and the International Conference on Medical Humanities further strengthened academic engagement and regional visibility. Notably, the Sri Lankan model emphasizes locally developed content grounded in indigenous cultural, religious, and artistic traditions, thereby offering a distinctive Southern Asian perspective on medical humanities and contributing to broader regional discourse [22].

#### The US

A study by Joynes in 2003, titled “Becoming a Humanities Curriculum: The Center for Humanities and Medical Ethics at the University of Texas Health Science Center at San Antonio,” aimed to develop and implement a longitudinal, integrated humanities curriculum at the School of Medicine at the University of Texas Health Science Center in San Antonio. The authors describe how this curriculum was created and implemented, including the history of ethics and humanities education at the school, which led to the founding of the Center for Humanities and Medical Ethics in July 2002. The core philosophy of the new curriculum focused on protecting, nurturing, and respecting students’ human nature, dynamic imagination, and individuality by leveraging the arts to foster critical thinking, value emotion, and model honest, respectful communication. This was achieved through the efforts of two full-time faculty members affiliated with the humanities—one a medical doctor with a degree in creative writing, and the other an English Ph.D. with a background in theater.

The curriculum aimed to help students grasp biomedical ethics and ethical analysis methods, encourage critical thinking, provide feedback, promote reflection, develop cultural sensitivity and self-awareness, enhance communication and active listening skills, and foster professional character and behavior. During the first year, students engaged with patient narratives, the basics of ethics, ethical decision-making, documentary films, and relevant case discussions for an integrated humanities education. In the second year, efforts were made to familiarize students with poetry and fiction through extracurricular activities such as a book club or study group. By the third year, a historical perspective and knowledge of related legends were introduced, and students also shared their narratives and memories [23].

#### Europe

A study titled “Special Study Unit in Medical Humanities on the Principles of Medical Theory and Practice” was conducted at Charité, Humboldt University, Berlin, Germany, by Kiessling et al. in 2003. The authors, members of the Curriculum Review Committee at the Medical Faculty of Humboldt University, highlighted the social aspects of the patient-physician relationship and the medical profession. The results indicated that integrating the humanities into medical education helps students become more aware of the strengths and weaknesses of modern medicine, develop a sense of responsibility, and broaden their overall perspective and worldview. The professors involved came from various disciplines, including the history of medicine, medical ethics, sociology, anthropology, and complementary medicine. Once a week, one or two professors taught a ninety-minute class to a group of twenty-one students. There was one seminar for every 12 sessions, and students attended four seminars during their five-year training [10].

Across European medical schools, the integration of MH has become increasingly institutionalized, yet remains heterogeneous in scope, structure, and epistemological orientation. A recent meta-review published in *Frontiers in Medicine* indicates that European curricula frequently incorporate philosophy, ethics, anthropology, history of medicine, narrative medicine, and diverse arts-based pedagogies, either as stand-alone modules or as longitudinal components embedded within professionalism and communication training. The dominant justification in Europe tends to be epistemological and competency-oriented: humanities are framed as a form of critical knowledge that strengthens clinical judgment, ethical reasoning, reflective capacity, and patient-centered communication. In addition, several programs emphasize resilience and burnout reduction through reflective writing and narrative practices [24].

In contrast to many Asian contexts—where medical humanities initiatives are often institution-driven, culturally grounded, and shaped by local religious and artistic traditions—European programs appear more structurally embedded within formal curricular frameworks and aligned with learning-outcome-based education models. However, similar to Asian experiences, European curricula display substantial variability in content, teaching hours, and assessment methods, and robust longitudinal evidence demonstrating sustained impact on professional behavior remains limited. Thus, while Europe provides a more formalized and epistemologically articulated integration of Medical Humanities, both regions continue to grapple with challenges related to standardization, evaluation, and long-term outcome measurement.

#### Canada

In Canada, advocacy is also regarded as a core competency for physicians, but it is considered challenging to teach. Researchers have proposed that health humanities could be a new tool for developing this skill. Recent reviews indicate that integrating health humanities into educational programs improves skills such as effective communication, self-reflection, and a patient-centered approach—qualities that support advocacy. The experience at Dalhousie University in Canada demonstrates that strategies such as decentralizing the physician as the sole expert, involving students in educational decisions, and incorporating narrative and art can enhance the objectivity of advocacy education in medicine [25].

#### Australia

In his article, published in the Review of Medical Humanities at the University of Melbourne, Matthew Melhem emphasizes that the growing incorporation of medical humanities into medical school curricula underscores its significance in cultivating physicians who adopt a patient-centered and culturally competent approach. The Professional Practice program at the University of Melbourne comprises modules including professional reflection, professional identity, medical ethics, and law. Nevertheless, the research suggests that student perceptions and attitudes may hinder the achievement of these objectives; a considerable number of students perceive the flexibility and uncertainty inherent in the humanities as less attractive compared to the definitive nature of biomedical sciences and exhibit diminished receptiveness to traditional humanities assessment methodologies, such as reflective writing. Concurrently, student involvement in co-facilitating classes has enhanced their communication and leadership abilities, although the extent of participation is influenced by individual circumstances. This experience illustrates that collaborative engagement between medical schools and students is vital for developing a curriculum that effectively complements and aligns with clinical training [26].

### Impact of humanities on medical students

Wald et al. conducted a study titled Medical Humanities in Medical Education and Practice in 2019. This study explores the nature and importance of medical humanities education. It examines the strengths in creative and intellectual areas of disciplines such as literature, art, creative writing, drama, film, music, philosophy, ethical decision-making, and anthropology, aiming to include these tools in medical education to help humanize clinical medicine. There has been much debate about how the medical humanities should be integrated into the medical curriculum. The role and necessity of incorporating medical humanities into areas like behavioral health, communication skills, cultural awareness, palliative care, and aging have been discussed. Humanities-based education can complement basic and clinical training. The growth of medical humanities, through collaboration with disciplines such as language, art, music, and history, can help make medicine more universal. Technology can support these educational goals. Medical humanities foster empathy and self-reflection among physicians, promoting their well-being, recovery, and resilience [5].

Blease et al. conducted a study titled Medical Humanities and Medical Education in 2016. The idea that studying the humanities helps to humanize physicians has become a popular topic within the field. It is argued that the humanities (especially literature) help to foster insights beyond those provided by biomedical education. It is claimed that young physicians can thus gain significant insight into patient care and develop important skills valuable for their professional lives. The study concludes that medical and health humanities, particularly literature, can help foster insights beyond those provided by classic biomedical science education. Young physicians assert that they can gain new perspectives about illness and the patient’s world and acquire skills that will assist them in their careers. This study highlights the intrinsic value of medical humanities in physician education and advocates for their integration into medical curricula. The researcher suggests that a medical humanities manifesto would be more intellectually honest and coherent and would better defend its value in medical education [27].

A study by Graham et al. in 2016 titled “Medical Humanities Courses Are Associated with Greater Empathy in Medical Students” examined the impact of medical humanities courses on students. In this research, students enrolled in an elective course in medical humanities for academic credit. The Jefferson Scale of Empathy Student Version (JSE-S) was administered at the start and end of an academic year during which humanities courses were offered. Changes in JSE-S scores for students who took the medical humanities course were compared to those who did not take any humanities courses. Empathy issues and reduced empathy are common challenges among physicians, evident throughout medical education. The study aimed to determine whether medical humanities courses could help address and improve these issues.

The results showed that elective medical humanities courses significantly enhanced students’ empathic abilities and markedly decreased empathy violations. Results indicated that medical humanities courses were associated with improved empathy outcomes among medical students. Among students who did not enroll in humanities courses, 71% either declined or showed no improvement in their JSE-S scores during the academic year. Conversely, 46% of students who took humanities courses either declined or showed no improvement in their JSE-S scores. This difference was statistically significant (*P* = .03). The medical humanities curriculum was associated with positive empathy outcomes as measured by the JSE-S. The study concluded that optional medical humanities courses were associated with improved empathy scores among a group of U.S. medical students. This could suggest a direct impact of the humanities courses. Alternatively, students’ choice to take medical humanities courses might signal a predisposition toward greater empathy development [28].

Several studies have also investigated the impact of medical humanities education on medical students’ professional and interpersonal development. A systematic review and meta-analysis published in *BMC Medical Education* found that MH programs significantly improved empathy among medical students and healthcare professionals, particularly when courses incorporated reflective writing and experiential learning approaches [29]. Studies have also reported that participation in humanities coursework, such as Poetry and Creative Writing, is associated with higher scores on validated empathy measures, suggesting that humanities exposure may help mitigate the decline in empathy often observed during medical training [30, 31]. In addition to empathy and compassion development, humanities-based educational interventions have been associated with improvements in communication skills, ethical reasoning, and reflective capacity, thereby contributing to the formation of professional identity and more patient-centered clinical practice. As a result, it also provides a greater sense of satisfaction in clinical encounters [32].

### Challenges, opportunities, and recommendations

Chiavaroli et al. (2012) conducted a study titled “Medical Humanities and the Perils of Curriculum Integration.” The rise of integration in modern medical curricula can be seen as a benefit for medical humanities, allowing them to incorporate their perspectives into the curriculum through specific strategies. This article presents an example of integrating humanities content into a medical course, questions the desirability of a solely integrated approach, and highlights the value of maintaining a separate and coherent discipline for medical humanities in medical education. The study discusses the integration of humanities educational content, noting that while an integrated approach has benefits, it also presents challenges. In modern medicine, the integration of subjects in a case-based format is a topic of great interest. The areas of integration include empathy, compassion, self-care, emotional understanding, understanding the patient’s perspective, communication skills, cultural competence, and professional services—each representing a key theme in medical humanities curricula worldwide [33].

A recent article examining the necessity and methods of integrating medical humanities at Mazandaran University of Medical Sciences highlighted challenges in Iran, including cultural issues, resistance to change, medical hegemony, a clinical-based and reductionist view, the absence of an approved curriculum, and overcrowding [34]. Since medical and health humanities are still relatively new fields, they face a global shortage of specialized faculty. Two key points emerge: first, the need to train instructors dedicated specifically to teaching medical humanities; second, to familiarize current humanities lecturers and clinical faculty with the principles and approaches of this discipline.

This can be achieved through refresher courses, conferences, and professional development programs, helping them deliver content more intentionally, engagingly, and in a human-centered manner. Moreover, the heavy workload of the medical curriculum leaves little space for additional courses, making it even more crucial to motivate students to participate in extracurricular humanities education. Despite resource and time limitations, the growing body of literature and textbooks in medical humanities can facilitate integration. Special attention to cultural issues is especially important, as content must be tailored to each society’s background.

While human-centered education is rooted in cultural contexts, it must ultimately aim to enhance physicians’ cultural and intercultural skills, since their responsibility is to care for all individuals regardless of culture, religion, skin color, or beliefs. Utilizing modern technologies—such as interactive learning tools, clinical simulations, and multimedia resources—offers opportunities to advance medical humanities education. Global experiences also demonstrate that community-based approaches, including service-learning and social projects, not only improve communication and management skills among medical students but also help rebuild social trust between physicians and patients. Additionally, fostering a humanistic and ethical culture among future physicians provides a unique opportunity to restore medicine to its value-driven, human-centered roots.

### Innovation and additional value

This study is among the first comprehensive narrative studies on the integration of health humanities into the medical curriculum nationwide in Iran, with a specific focus on Mazandaran University of Medical Sciences.

According to the curriculum approved by the Ministry of Health in the Islamic Republic of Iran, the general courses specified in the curriculum are the same for many medical schools across Iran by March 2026. In this regard, this study also provides insights beyond the University of Mazandaran into medical education across Iran.

Previous studies, both nationally and internationally, have generally addressed the expanded role of the humanities in medicine or called for the addition of independent courses. Still, few have examined the systematic integration, organization, and framing of medical humanities in undergraduate curricula.

This review fills an important gap in the literature by shedding light on local challenges and international best practices. Its findings provide practical guidance for policymakers as well as curriculum developers, including the need for coordinated faculty development, institutional support policies, and culturally appropriate content. These developments will not only help Iranian medical schools meet international standards but also provide evidence-based strategies for improving empathy, communication skills, and cultural competence among future physicians.

The study will also help medical schools worldwide understand the importance of and how to incorporate health humanities into their curricula, taking action to achieve this goal.

## Conclusion

This narrative scoping review shows that integrating medical humanities into medical education in Iran, especially at Mazandaran University of Medical Sciences, is still in its early stages and lacks the systematic, comprehensive approaches found in top international institutions. The results confirm that intentionally including the humanities can enhance empathy, self-reflection, communication skills, and cultural competence in future doctors, thereby improving healthcare quality.

This study is one of the first studies to comprehensively map the current national situation and compare it with international experiences. It fills a significant gap in the literature and offers a clear guide for policymakers, curriculum designers, and educational developers. Emphasized priorities include institutional support, specialized faculty training, and adapting content to fit cultural contexts.

Ultimately, integrating global insights with Iran’s local context could create a new model for medical education that stays true to the humanistic and value-based origins of medicine while adapting to international trends. This approach not only improves medical training but also helps restore social trust between doctors and patients.

### Limitations

This narrative scoping review has several limitations that should be acknowledged. First, the study employed a narrative rather than a systematic review approach; therefore, despite efforts to ensure comprehensive coverage, some relevant studies may not have been identified or included. Second, the search was limited to selected international databases and Persian literature sources, which may have resulted in the omission of relevant publications from other regions, particularly some Asian and developing countries. Third, variations in terminology, curricular structures, and reporting standards across institutions made direct comparisons challenging. Additionally, the analysis relied primarily on published literature and publicly available curricular information rather than primary institutional data, which may limit the depth of contextual interpretation. Finally, as the medical humanities is an evolving interdisciplinary field, rapid developments in curricula and educational policies may not yet be fully reflected in the literature reviewed.

## Data Availability

All data generated or analyzed during this study are included in this published article.
